# Giant infiltrative myxopapillary ependymoma of filum terminale with sacral erosion: a case report and literature review

**DOI:** 10.1097/MS9.0000000000005167

**Published:** 2026-05-14

**Authors:** Ali Mehdi, Hassan Mehdi, Sabahat Batool, Shayaan Pervez, Faiz Ur Rehman, Muhammad Idress Karwan, Asma Waheed, Ehsan Ullah Akrami

**Affiliations:** a Medicine Department, PHQ Teaching Hospital, Gilgit, Pakistan; b Department of Neurosurgery, Nishtar Medical University and Hospital Multan, Pakistan; c Fatima Jinnah Medical University, Sir Gangaram Hospital Lahore, Pakistan; d Department of Oncology, Nishtar Hospital Multan, Pakistan; e Mawlana Jalaluddin Mohammad Balkhi University, Afghanistan

**Keywords:** adjuvant radiotherapy, case report, filum terminale, myxopapillary ependymoma, spinal cord neoplasms

## Abstract

**Introduction and importance:**

Myxopapillary ependymoma (MPE) is a rare neuroepithelial tumor predominantly localized to the filum terminale. While traditionally viewed as indolent, its reclassification to World Health Organization (WHO) Grade 2 in 2021 reflects a significant risk of recurrence and neuraxial dissemination. Giant infiltrative variants (>10 cm) with sacral erosion are exceptionally rare and present formidable surgical and oncological challenges.

**Presentation of case:**

A 30-year-old female presented with a 2.5-year history of progressive radiculopathy, culminating in paraparesis (MRC Grade 2/5), urinary and fecal incontinence. Neuroimaging revealed a massive, heterogeneously enhancing intradural mass (19 × 8.6 × 6.3 cm) extending from the conus medullaris (L1) to the sacrum, demonstrating significant sacral erosion, foraminal expansion, and presacral extension. The patient underwent an L3–L4 laminectomy for decompression and tumor reduction. Due to dense adherence to the cauda equina, a subtotal resection (approximately 30–40%) was performed to preserve neurological integrity. Histopathology confirmed WHO Grade 2 MPE with a low Ki-67 index (<2%). Postoperative residual disease was managed with adjuvant external beam radiotherapy (50 Gy in 25 fractions). At 5 months, the patient regained independent ambulation with significant motor recovery and achieved complete resolution of sphincter dysfunction.

**Clinical discussion:**

The extent of resection remains the primary prognostic indicator; however, in “giant” infiltrative cases, gross total resection is often precluded by dense neural adherence. In such scenarios, subtotal resection combined with adjuvant radiotherapy (50–54 Gy) is crucial for achieving local control and facilitating neurological recovery. The aggressive local destruction observed here, despite a low proliferative index, underscores the unpredictable nature of MPE.

**Conclusion:**

Giant MPEs can exhibit locally aggressive behavior and significant anatomical destruction. A multidisciplinary approach utilizing strategic subtotal resection, adjuvant radiotherapy, and rigorous longitudinal surveillance is vital for managing extensive lesions while prioritizing functional outcomes.

## Introduction

Myxopapillary ependymoma (MPE) is a distinct spinal ependymoma arising predominantly from the filum terminale and cauda equina[1]. In the 2021 World Health Organization (WHO) Classification of Tumors of the Central Nervous System, MPE was reclassified from Grade 1 to Grade 2 due to its recognized recurrence potential and risk of neuraxial dissemination[1]. The annual incidence is approximately 0.05–0.08 per 100 000 population, most commonly affecting young adults[2]

HIGHLIGHTS

- Report of a giant 19-cm infiltrative myxopapillary ependymoma with significant sacral bone erosion.
- Demonstrates aggressive local anatomical destruction despite a low Ki-67 proliferative index (<2%).
- Strategic subtotal resection successfully preserved cauda equina function despite dense neural adherence.
- Adjuvant radiotherapy (50 Gy) provided effective early local control and facilitated significant neurological recovery.

Clinically, MPE presents with chronic low back pain and radiculopathy owing to its slow growth. Neurological deficits and sphincter dysfunction tend to occur late, frequently resulting in delayed diagnosis[3]. Magnetic resonance imaging is the diagnostic modality of choice and usually demonstrates an intradural extramedullary lumbosacral mass with contrast enhancement[4].

Histopathologically, MPE is characterized by papillary structures composed of cuboidal to columnar tumor cells arranged around hyalinized fibrovascular cores embedded within a myxoid (mucin-rich) stroma. Tumor cells typically express glial fibrillary acidic protein (GFAP) and S100 on immunohistochemistry, confirming glial differentiation^[^1,5^]^. Despite its low proliferative index and relatively bland cytology, increasing evidence suggests that histologically low-grade MPE can exhibit infiltrative growth patterns, capsular disruption, and aggressive local extension, contributing to recurrence risk[5]

The extent of resection remains the most important prognostic factor, with gross total resection associated with improved progression-free survival (PFS) compared to subtotal resection. However, complete excision may not be feasible in infiltrative cases involving neural structures. In such scenarios, adjuvant radiotherapy is often considered to improve local control^[^6,7^]^

We report a giant (19 cm) infiltrative MPE with sacral erosion, managed with subtotal resection followed by adjuvant radiotherapy, highlighting the potential for aggressive local behavior despite low-grade histology. This case is reported in accordance with the SCARE guidelines[8].

## Case presentation

This case was managed at Nishtar Medical University & Hospital, Multan, Pakistan, between September and November 2025. A 30-year-old unmarried woman of South Asian ethnicity, unemployed and living with her family, presented to the neurosurgical outpatient department on 15 September 2025 with a 2.5-year history of right-sided leg pain radiating to the lower back. Over the preceding 5 months, she had progressively lost the ability to stand or walk independently. Three weeks prior to presentation, she had developed bilateral lower-limb pain and weakness associated with urinary and fecal incontinence.

She had no history of hypertension, diabetes mellitus, allergies, or regular medication use apart from occasional analgesics. There was no history of prior hospital admissions, spinal surgery, trauma, malignancy, or chronic medical illness. She had no previous record of neurological evaluation for her symptoms.

## Clinical assessment

On examination, the patient was alert and oriented. Motor examination revealed severe bilateral lower-limb weakness with Medical Research Council (MRC) grade 2/5 in both lower limbs, while upper-limb strength was normal (MRC 5/5 bilaterally). Sensory examination was intact. Muscle tone was increased, and deep tendon reflexes were exaggerated in the lower limbs. There was no muscle wasting or palpable mass. Rectal examination revealed reduced anal sphincter tone. The remainder of the systemic examination was unremarkable. Plain radiographs of the lumbosacral spine were unremarkable.

## Radiological assessment

**Figure 1.**
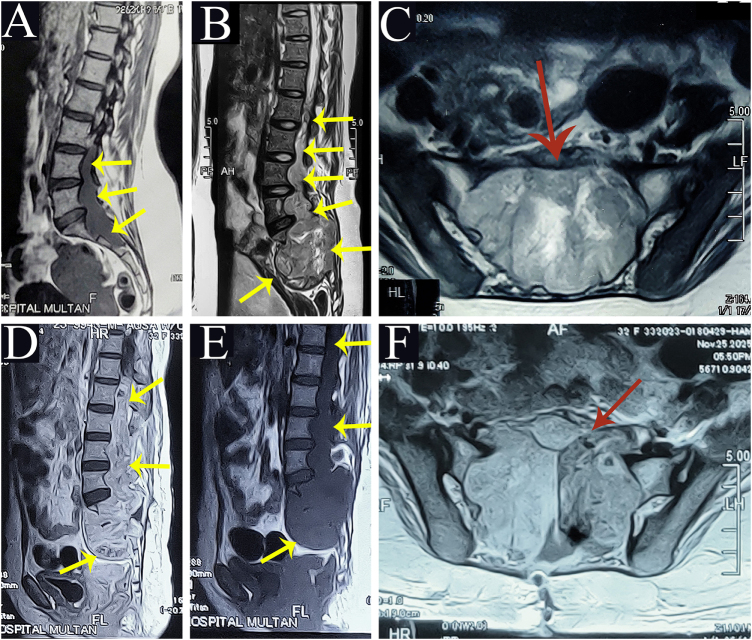
MRI of the lumbosacral spine demonstrating a giant myxopapillary ependymoma and postoperative status. (A) Sagittal T2-weighted non-contrast MRI showing an infiltrative lesion extending from L3 to S2. (B) Sagittal T2-weighted non-contrast MRI demonstrating marked progression with a large multilobulated mass extending from the conus medullaris (L1) to the sacrum. (C) Axial T2-weighted MRI showing sacral involvement and widening of the neural foramina. (D–E) Sagittal T1-weighted post-contrast MRI demonstrating decompression of the thecal sac with residual enhancing tumor. (F) Axial T1-weighted post-contrast MRI showing persistent sacral and foraminal involvement. Yellow arrows indicate tumor extent and thecal sac expansion, while red arrows highlight neural foraminal extension and sacral involvement.

Initial magnetic resonance imaging (MRI) (Fig. 1A), sagittal non-contrast MRI of the lumbosacral spine obtained approximately 2 years prior to presentation, demonstrates an infiltrative abnormal signal intensity lesion extending from L3 to S2, measuring approximately 11.1 cm in craniocaudal length and 2.5 cm in anteroposterior dimension. The lesion is associated with posterior vertebral body scalloping and mild expansion of the thecal sac, suggesting a slowly progressive intradural mass.

Preoperative MRI of the lumbosacral spine (Fig. 1B–C) demonstrates a giant infiltrative intradural mass. Sagittal (B) and axial (C) non-contrast MRI images obtained on 17 September 2025 show a large multilobulated infiltrative mass extending from the conus medullaris (L1) to the sacrum, measuring approximately 19 × 8.6 × 6.3 cm (craniocaudal × transverse × anteroposterior). The lesion appears hyperintense on T2-weighted imaging and is associated with marked expansion of the thecal sac. There is involvement of the sacral vertebrae, sacral alae, and bilateral L5–S3 neural foramina, with widening of the foraminal canals. The tumor extends into the presacral space, with close proximity to the rectum and adjacent musculature. Associated sacral and L5 vertebral body erosion is also noted.

Contrast-enhanced sequences were not available at the time of preoperative imaging due to technical limitations. However, the non-contrast MRI adequately demonstrated the lesion extent, spinal canal compromise, and osseous involvement.

Differential diagnoses included MPE, chordoma, paraganglioma, and other infiltrative neoplasms.

Given the extensive osseous involvement, a Technetium-99m methylene diphosphonate (99mTc-MDP) bone scan was performed to exclude distant metastasis. However, no scintigraphic evidence of metastatic disease was identified.

Lumbar puncture was deferred due to the near-complete effacement of the subarachnoid space and the risk of neurological deterioration. Computed tomography was not performed, as MRI sufficiently demonstrated sacral erosion and provided adequate anatomical detail for surgical planning.

## Surgical management

On 25 September 2025, the patient underwent an L3–L4 laminectomy and decompressive tumor resection performed by the neurospine surgery team.

Intraoperatively, a gelatinous, moderately vascular tumor densely adherent to the cauda equina nerve roots was encountered. Due to extensive neural adherence, a strategic subtotal decompressive resection was performed to preserve neurological function. Approximately 30–40% of the tumor bulk was removed. Portions of the capsule firmly adherent to functional nerve roots were intentionally left *in situ*.

Intraoperative neuromonitoring was unavailable; therefore, meticulous microsurgical dissection with visual nerve root preservation was employed. No plastic surgical involvement was required, as primary wound closure was achieved without difficulty.

## Immediate postoperative status

Postoperatively, motor strength remained at MRC grade 2/5 in both lower limbs, with no new neurological deficits. Sensory function remained intact, and no worsening of bowel or bladder dysfunction was observed.

The patient remained hospitalized for 5 days and was discharged in stable condition, with a referral to the oncology department for adjuvant radiotherapy.

## Histopathological findings

**Figure 2.**
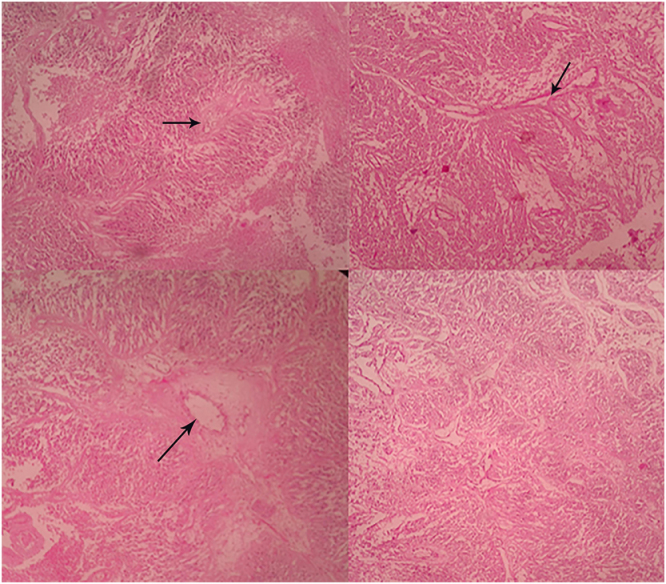
Histopathological features of myxopapillary ependymoma. Hematoxylin and eosin–stained sections demonstrate characteristic papillary architecture composed of cuboidal-to-columnar tumor cells arranged radially around hyalinized fibrovascular cores embedded in a myxoid (mucin-rich) stroma. In the upper panels, arrows indicate papillary structures with central hyalinized fibrovascular cores, a hallmark feature of myxopapillary ependymoma. Tumor cells are arranged in a perivascular pattern, forming pseudopapillary projections into the surrounding myxoid matrix. In the lower left panel, the arrow highlights a dilated vascular channel within a hyalinized fibrovascular core, surrounded by tumor cells and abundant myxoid stroma.

Histopathological examination of the excised tumor tissue in Figure 2 demonstrated characteristic features consistent with MPE. Hematoxylin and eosin–stained sections revealed papillary structures composed of cuboidal to columnar tumor cells arranged radially around hyalinized fibrovascular cores embedded within an abundant myxoid (mucin-rich) stroma. The tumor cells displayed uniform round-to-oval nuclei with finely granular chromatin and inconspicuous nucleoli, with moderate amounts of eosinophilic cytoplasm. The papillary architecture and perivascular arrangements were prominent, forming pseudopapillary projections surrounding vascular cores, which is a characteristic histological feature of MPE. Areas of stromal myxoid degeneration and hyalinization of vascular walls were also observed. No significant nuclear atypia, necrosis, or microvascular proliferation was identified. Immunohistochemical analysis demonstrated strong positivity for GFAP and S100 protein, confirming glial differentiation. The Ki-67 proliferation index was low (<2%), supporting the relatively low proliferative activity of the tumor.

These histopathological and immunohistochemical findings confirmed the diagnosis of WHO Grade 2 MPE. A separate bone biopsy was not performed, as imaging and histopathology were consistent with direct tumor invasion.

## Postoperative imaging

Postoperative contrast-enhanced MRI of the lumbosacral spine (Fig. 1D–F) demonstrates residual infiltrative tumor following subtotal resection. Sagittal (D–E) and axial (F) contrast-enhanced MRI images obtained on 25 November 2025 demonstrate adequate decompression of the thecal sac following L3–L4 laminectomy and partial tumor resection. A residual heterogeneously enhancing infiltrative soft tissue mass measuring approximately 20.9 × 7.0 × 6 cm (craniocaudal × transverse × anteroposterior) is seen extending from D12 to the sacrum, with involvement of the sacral vertebrae and neural foramina. Persistent foraminal widening and sacral involvement are evident. Linear leptomeningeal enhancement extending up to D11 is also noted.

## Adjuvant therapy

Given residual disease following subtotal resection, the patient received adjuvant external beam radiotherapy using a Cobalt-60 unit. A total dose of 50 Gy in 25 fractions was delivered over 4.5 weeks (five fractions per week).

## Follow-up

At follow-up on 26 February 2026 (five months postoperatively), motor strength had improved to MRC grade 4/5 in both lower limbs. The patient was able to ambulate independently without assistance. Urinary incontinence resolved completely, and anal sphincter tone was restored. Ongoing surveillance has been instituted, given the potential risk of neuraxial dissemination.

## Discussion

Although MPE is generally indolent, aggressive local invasion has been increasingly recognized[5]. Our case demonstrated MPE causing sacral bone erosion and extensive foraminal extension despite a low proliferative index.

Extent of resection is the most consistently reported prognostic factor in MPE. In the literature, recurrence rates are approximately 13–15% following gross total resection, compared with up to 30–45% following subtotal resection, particularly in cases with capsular violation[9]. In a multi-institutional study with long-term follow-up, significantly improved local control and PFS were demonstrated in patients undergoing complete excision[6]. However, these data are derived primarily from retrospective analyses with heterogeneous radiotherapy use and variable follow-up durations, limiting direct comparison and underscoring the need for prospective registries.

Contemporary population-based analyses confirm that STR is independently associated with an increased recurrence risk[4]. Given the dense neural adherence in our case, laminectomy with subtotal resection was prioritized over aggressive excision.

Although doses between 45 and 54 Gy are commonly reported, available evidence does not consistently demonstrate superior outcomes with dose escalation beyond conventional ranges. Published data suggest improved PFS with adjuvant radiotherapy following subtotal resection, particularly in the 50–54 Gy range^[^10,11^]^. Therefore, a total dose of 50 Gy in conventional fractionation was selected in our case to optimize local control while remaining within established spinal cord tolerance constraints, as outlined by QUANTEC recommendations[12]. Potential late complications of spinal radiotherapy include radiation-induced myelopathy, radiculopathy, vertebral compression fractures, secondary malignancy, and pelvic organ dysfunction^[^10,11^]^.

Postoperative MRI confirmed residual disease, supporting the indication for adjuvant therapy. In our case, the tumor’s large size, extension through foramina, invasion of the sacrum, and bone erosion were unusual and could resemble more aggressive tumors, making diagnosis and surgical planning more difficult.

Large lumbosacral masses with bone erosion may mimic schwannoma, chordoma, paraganglioma, or metastasis[5]. Histopathological confirmation remains essential.

To contextualize our findings, we performed a literature review and included 12 cases that reported large MPEs (Table. 1). Patient demographics, tumor characteristics, surgical approach, adjuvant therapy, and clinical outcomes were analyzed.

**Table 1:** Literature review of reported cases of large myxopapillary ependymoma.

Author (year)	Age/sex	Tumor location	Clinical presentation	Tumor size	Surgical management	Radiotherapy	Follow-up & outcome
Arjomand *et al.* (2025)[13]	48/M	T12–L1	Intermittent back pain (2 years), urinary urgency, fecal incontinence.	Not specified (enhancing mass)	Gross total resection	Recommended but patient declined.	Stable urinary incontinence at first post-op visit; long-term monitoring planned.
Balodis *et al.* (2024)[5]	44/M	L1–S4 (Giant) + Brain/Spine Metastases	Non-specific low back pain (2 years).	56 × 96 × 190 mm (Giant)	Partial resection	Adjuvant RT administered (due to metastases).	1 year: tumor growth and new metastases; suboptimal outcome.
Achoura *et al.* (2024)[14]	32/M	L2–L3 (intradural extramedullary)	Bilateral lumbocrural pain, limited walking (50 m) for 6 months.	13 × 17 × 24 mm	Gross total resection	Not administered (Total resection).	Discharged day 2; evolved well.
Kouhen *et al.* (2024)[15]	68/M	Sacrum (endocanal)	Persistent left low back pain radiating to legs, fecal dysfunction (3 mos).	Not specified (expansile mass)	Subtotal excision	Adjuvant VMAT (59.4 Gy).	18 months: No clinical signs of residual tumor; no deficits.
Almatrafi *et al.* (2023)[16]	28/M	Multifocal: T12–L3 (conus/filum) + L4, L5, and nerve roots	Low back pain (1 year), radiating left leg pain, perianal anesthesia.	Large (T12–L3)	Surgical resection	Not administered (Surgery only).	Post-op MRI: no tumor progression; marked symptom improvement.
Caporalini (2022)[16]	16/M	Multifocal: L5–S2 and L2–L3	Mechanical back pain; no neurological deficits.	6 cm (caudal); 1.5 cm (rostral)	Subtotal (caudal) + total (rostral)	Craniospinal RT (50.4 Gy total) due to multifocal nature.	6 months: no recurrence; reduction of residual caudal mass.
Tabor *et al.* (2022)[17]	34/M	Multifocal: L1–L3 and S1–S2	Painful radiculopathy, sexual dysfunction, altered defecation.	Not specified (large conus lesion)	Staged resection (subtotal)	Focal IMRT (54 Gy) to L1–S2.	6 months: pain resolved; persistent sexual dysfunction.
Lien *et al.* (2021)[18]	24/F	Right gluteal region (extra-CNS)	Gluteal pain worsened by sitting; palpable mass.	3.7 × 1.8 × 3.3 cm	*En bloc* resection (gross total)	Deferred (*en bloc* resection achieved).	6 months: no recurrence or metastatic disease.
Omerhodžić *et al.* (2020)[19]	33/M	Multifocal: L1–L2, L3, S1	Sudden urinary retention.	Large cystic (L1–L2)	Total Resection (all 3 tumors)	Not administered (total resection of all 3 tumors).	Complete recovery; urinary retention resolved.
Marchesini *et al.* (2019)[20]	23/M	T1–T12 (giant)	Progressive paraparesis, numbness, urinary hesitancy.	T1–T12 (giant)	Subtotal resection	Not administered.	2.5 years: stable residue; almost complete motor recovery.
Pusat *et al.* (2018)[21]	36/F	T11–S1	Back pain radiating to legs (5 years), progressing to paraparesis.	~20 cm	Total resection	Not necessary (total resection).	2 years: mobilized without support; no residual lesion.
Khan *et al.* (2017)[22]	32/M	Multifocal: L1–L2, L3, S1	Progressive incontinence (2 years), bladder areflexia.	Large (L1–L2)		Not administered.	6 months: disease free; neurologically intact.

This single-case report has limited generalizability. While follow-up is currently limited to 5 months, long-term surveillance is ongoing, and future updates will be necessary given the known risk of late recurrence. Resource constraints limited intraoperative neuromonitoring, early postoperative imaging, and CSF analysis are limitations to this study.

Future multicenter registries with standardized imaging protocols and long-term follow-up (≥5–10 years) are needed to better define recurrence risk and optimize treatment strategies. Prospective registries incorporating standardized postoperative MRI surveillance and long-term neurological outcome assessments would help clarify optimal management strategies.

Clinicians should recognize that even histologically low-grade MPEs may demonstrate locally aggressive behavior. Treatment strategies should, therefore, prioritize maximal safe resection, appropriate adjuvant therapy in cases of residual disease, and structured long-term surveillance.

Recent bibliometric analyses highlight the rapidly expanding role of large language models, such as ChatGPT, in medical research and academic writing, emphasizing the importance of transparency, ethical oversight, and appropriate human verification when integrating artificial intelligence–assisted tools in scientific publications[23].

## Conclusion

MPE, although histologically low-grade, may demonstrate aggressive local invasion. Subtotal resection, radiological confirmation of residual disease, appropriate adjuvant radiotherapy, and long-term structured surveillance are essential to optimize neurological and oncological outcomes.

## Data Availability

The data supporting the findings of this study are available from the corresponding author upon reasonable request.

## Concurrent submissions

This case is not under consideration elsewhere.
